# Advanced modular automated calculation of the morpho-histological parameters in myocardial infarction

**DOI:** 10.15190/d.2016.13

**Published:** 2016-09-30

**Authors:** Adelina Curaj, Lucian Pop-Fele, Marko Jovanovic, Stephan Michael Jonas, Julia Moellmann, Doina Ghertescu, Ovidiu Constantin Novac, Mihaela Rusu, Elisa A. Liehn

**Affiliations:** Institute for Molecular Cardiovascular Research (IMCAR), RWTH Aachen University, Germany; Institute for Molecular Cardiovascular Research, University Hospital, RWTH Aachen, Germany; Department of Medical Informatics, RWTH Aachen, Germany; Faculty of Electrical Engineering and Information Technology, University of Oradea, Oradea, Romania; Department of Cardiology and Pulmonology, Medical Faculty, RWTH Aachen University, Germany; Cardiology Department, University of Medicine and Pharmacy, Targu Mures, Romania; IZKF, Aachen, RWTH Aachen, Germany; Human Genetic Laboratory, University of Medicine and Pharmacy, Craiova, Romania

**Keywords:** Advanced modular automated calculation, myocardial infarction, infarction size estimation, 3D reconstruction

## Abstract

Myocardial infarction represents the most investigated pathology. Heart tissues are histologically assayed on a routine base in clinical laboratories. However, the lack of a standard operation procedure for e.g. calculating the size of the infarcted area of myocardium, may lead to an increased errors’ interval, with little correlation between results of a same tissue and/or with other pathophysiological parameters. This creates a clear barrier for further development of novel therapeutic strategies. In the present study, we present the robust applicability of a novel methodology such as: advanced modular automated calculation of (i) the size of infarcted heart of mice, (ii) the net collagen content present in the scar, and (iii) the interstitial fibrosis in remote, which are simultaneously performed. The new approach of defining the infarct size, one of the important predictor of every cardiac therapeutic intervention, will create a positive impact in the research accuracy. Additionally, it will be expected that the readily assembled reports of simultaneously computed parameters and its user-friendly operation allow an efficient and effective estimation of measurements.

## Introduction

Myocardial infarction represents the most common cause of death in the world. Therefore, huge efforts are made to investigate the patho-physiological mechanisms of the initiation, progression, and healing of heart after the acute myocardial infarction^1^. Likewise, different tissue regenerative strategies are proposed aiming to service patients suffering from myocardial infarction^2^. Despite all effort from last decades, the outcome results are still controversial, delimitating the research towards a valuable, accessible therapeutic strategy.

Heart tissues are histologically assayed on a routine base in clinical laboratories. However, the interpretation of processed immunohistochemistry images relies on the expertise of pathologist, which is subjective. Moreover, the lack of a standard operation procedure for, e.g. calculating the size of the infarcted area of myocardium, may lead to an increased errors’ interval, with little correlation between results of a same tissue and/or with other pathophysiological parameters. This creates a clear barrier for further development of novel therapeutic strategies. The conventional methodology for estimating the infarcted area usually considers at least three random tissue sections throughout the infarcted region, followed by histological staining (e.g. Masson Thrichrom, Hematoxylin Eosin). The localization and estimation of the infarcted size is made by using dedicated software^3^. Usually, this method is applied for large animals (rats, rabbits) in addition to functional studies such as: the assessment of serum markers, technetium-99m sestamibi single-photon emission computed tomography (SPECT) myocardial perfusion imaging or magnetic resonance imaging^4^. However, the latter will become more expensive to be applied in small animal models.

Nowadays, computer assisted methodologies are conducted to statistically estimate the size of the infarcted area for large number of serial sections Nowadays, computer assisted methodologies are conducted to statistically estimate the size of the infarcted area for large number of serial sections throughout the entire heart^5,6^. Alternatively, a single step midline length measurement approach^7^ delineates the infarcted area from which its size is estimated. However, this method is susceptible of errors when the infarction is not transmural and the differences obtained by the therapeutic effects are insignificant between the groups. The calculation of the infarction size from the entire heart volume requires extensive work including staining, image processing, measurements and calculations. Although image analysis and processing are assisted by specialized software, yet they still require manual interaction that can lead to subjective measurements. To our knowledge, commercial software does not enable the fully automatic calculation of the infarction size of each tissue section.

Here, we present the robust applicability of a novel methodology named advanced modular automated calculation (AMAC) to simultaneously calculate (i) the size of infarcted heart of mice, (ii) the net collagen content present in the scar, and (iii) the interstitial fibrosis in remote. The new approach of defining the infarct size- one of the important predictor of every cardiac therapeutic intervention will create a positive impact in the research accuracy. Additionally, it will be expected that the readily assembled reports of simultaneously computed parameters and its user-friendly operation allow an efficient and effective estimation of measurements.

## Methods

*Animal model of myocardial infarction*. Eight week-old male C57Bl/6 wild-type (Charles River, Germany) mice, underwent chronic myocardial infarction, as previously described^8^. Briefly, mice were intubated under general anesthesia using 100 mg/kg ketamine, 10 mg/kg xylazine, i.p. and ventilated with oxygen using a mouse respirator (Harvard Apparatus, Germany). After exposing the hearts by left thoracotomy, the left anterior descending artery was occluded using 0/7 silk. The ribs, muscle layer and skin incision were closed, and Buprenophin (0.1 mg/kg) was administrated until full recovery. The hearts were excised at four weeks, fixed in formalin and embedded in paraffin. All animal experiments were performed after obtaining the required permissions in accordance with European and German animal protection legislation.

*Histological morphometry*. The infarcted heart was sectioned from apex to mitral valve, and tissue sections were collected at every 300 µm, stained with Gomori’One Step Thrichom and imaged using the available microscope (Olympus). The total area of the heart tissue and the infarcted area were manually measured using both DISKUS (Hilgers, Germany) and AMAC. In order to compare the outcome results of the two methods, the data were expressed in pixel. The results of the first method represented the average values of overall infarcted areas of consecutive tissue sections, while the second method is based on the volumetric reconstruction of the serial sections into a 3D fashion, based on the following formula:

$$
S_{infraction} (\%) =  \frac{ \sum_{ \vartheta = 2}^{10}{( \vartheta * n)} }{ \sum_{ \theta = 2}^{10}{(  \theta  * n)} } *100
$$

where, *J *is the averaged infarction size over 10 samples, while *q* represents the total heart area, and *n* is the maximum number of pixels corresponding to a distance of 300 µm between sections. Furthermore, the infarction area was manually sectioned from the remote area. The blue color pixels corresponding to the presence of net collagen content in the scar was calculated and expressed as percentage. The same algorithm based on the red color definition of the pixels assigning the interstitial fibrosis existing in remote was accordingly assessed. The net collagen content and the interstitial fibrosis (%) were measured using both ImagePro and AMAC.

*Software development*. The software was implemented in Microsoft .NET technology with an integrated development environment (Visual Studio 2015 Community Edition, Microsoft). The application is designed to measure the infarct vs. healthy areas and also the percentage of collagen content and the interstitial collagen from a given heart section image. An image is a matrix of pixels, each pixel being defined by 3 color intensities: red, green, blue (RGB). The developed algorithm is counting the RGB values of every pixel within a well-defined threshold scheme. The scheme simultaneously defines the infarcted and healthy zone, as well as collagen from the given image based on their corresponding RGB values. Ten to fifteen applications are analyzed and reported in 10 seconds and returns a segmentation of each image into the different components. Thus, the accuracy of the results can visually be investigated. Summary parameters can directly be exported into Microsoft Excel format for further analysis. The segmentation images have a transparent background, so they can be easily reconstructed into a 3D Model.

## Results

**Figure 1:**
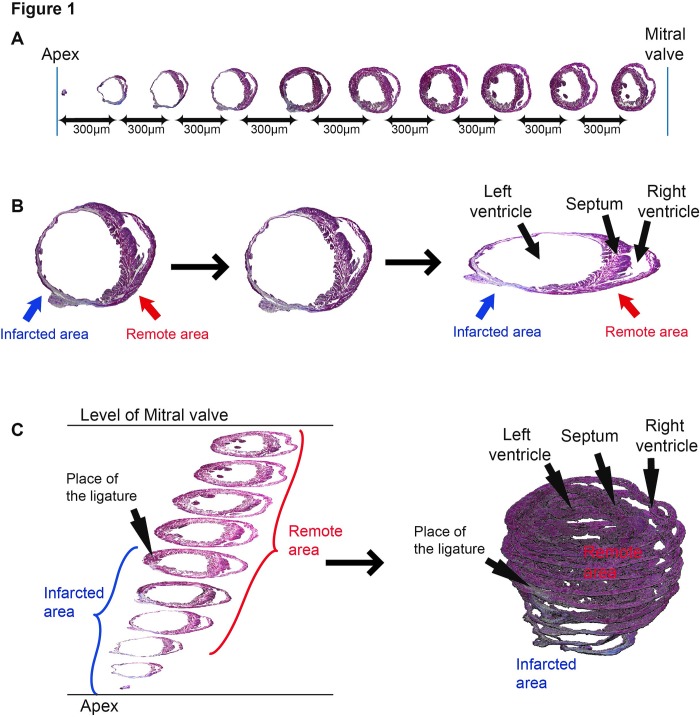
Assessment of infarction size using the AMAC software. [**A**] 10 serial sections, 300 µm apart, from apex to mitral valve are analyzed. [**B**] The digitalizing of the original image and [**C**] the 3D Reconstruction of the heart, visualizing the infarcted areas (blue), left ventricle, septum and right ventricle (red), as well as the placement of the ligature.

*Assessment of infarction size*. A comparison between the manual and computer measured infarction areas was performed. 10 serial sections from one 4 weeks infarcted heart were imaged and analyzed (**Figure 1** [A]). The Olympus software application converted each image in a digital mode, which precisely reproduced the original image (**Figure 1** [B]). After analyzing the pixels and the respective colors (blue and red), a report was provided. If desired, the images can be used for further 3D volumetric reconstruction (**Figure 1** [B,C]). After 3D reconstruction, the infarcted area could be visualized, as well as the place of the ligature. Thus, the reproducibility of the operative procedure can be monitored and controlled. The 3D reconstruction offers the possibility to estimate further cardiac parameters, such as dimensions of the left ventricle, septum and right ventricle (**Figure 1** [C]). The results showed that manually, a significant human error was involved by overestimating both total heart and infarction areas (**Table 1**). Our computer program, AMAC, was able to analyze each pixel, sorting it accurately within the color threshold range, thus eliminating the subjectivity and increasing the accuracy of the results.

Moreover, the computer program recognized even the hyperfine structures of interstitial fibrosis, which is present in the upper sections (7,8,9,10), but was completely ignored by the human eye.

The differences between the infarction sizes calculated in the conventional way (average from all infarcted areas) were significant, considering the small variations occurring during a therapeutic intervention^6^.

**Figure 2:**
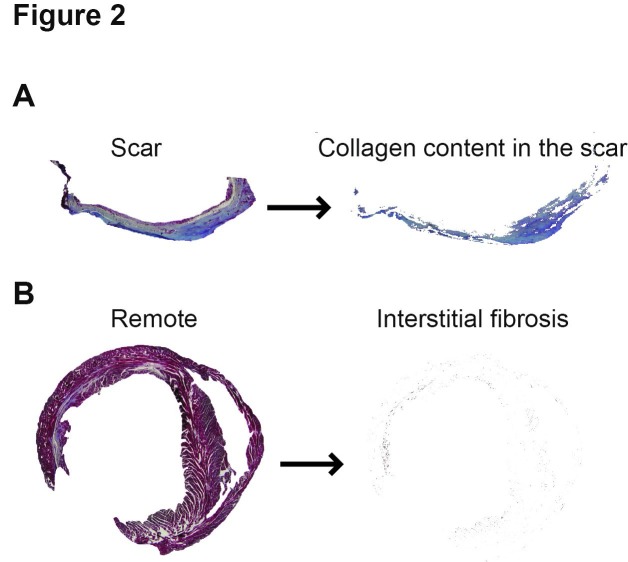
Assessment of the collagen content of the scar and the interstitial fibrosis. [**A**] The blue, dark blue pixels representing the collagen content in the scar, [**B**] as well as the interstitial fibrosis are assessed by AMAC

*Assessment of collagen content*. In the same sections, the collagen content was assessed in the scar (**Figure 2** [A]) by setting the threshold manually in ImagePro, and automatically with AMAC. In the remote area, the interstitial fibrosis was also assessed by the two methods (**Figure 2** [B]).

**Table 1:** Comparison between the manually assessment of infarcted area and AMAC.

		Manual				AMAC	
No.	Total heart area (px)	Infarcted area (px)	Infraction size (%)		Total heart area (px)	Infarcted area (px)	Infraction size (%)
1	-	-	-		-	-	-
2	79048	43023	**54,4**		65841	21610	**32,82**
3	149106	45402	**30,4**		103116	18666	**18,1**
4	214502	43172	**20,1**		174738	16941	**9,7**
5	338169	61102	**18,1**		275569	27678	**10,04**
6	433953	29029	**6,7**		338801	17933	**5,29**
7	462939	0	**0,0**		353645	11271	**3,19**
8	479865	0	**0,0**		372602	16221	**4,35**
9	470169	0	**0,0**		381734	13290	**3,48**
10	476956	0	**0,0**		369464	15220	**4,12**
Average of infarction area (%)			14,4				10,1
Volume infarction (%)		7,1				6,5	

The measurement in the collagen content between the manually and computer-assessed method differed vastly (**Table 2**), due to the overestimation of the blue colored area performed by the investigator as evaluated by visual inspection. Moreover, the investigator had to choose the color threshold for every section. The computer program was able to recognize different spectra of red and blue colors, thus the human error decreased significantly.

## Discussion

In this study, we demonstrate the feasibility of the AMAC software to recognize specific staining routinely used for histo-morphometry in the experimental model of myocardial infarction. Compared to manual assessment of the infarction size, AMAC eliminates the human error by accurately computing the infarction size. This is essential for proper interpretation of processed images, considering that the infarction in mousemodel is small and the variation between hearts, even operated by the same surgeon can be significant. Moreover, by 3D reconstitution of the heart, the proper placement of the ligature can be controlled, which is omitted by many researchers. If the ligature is not produced at the same level, the mouse should be excluded from the analyzed cohort to improve the accuracy of the results.

**Table 2:** Comparison between the collagen content and interstitial fibrosis assessment by ImagePro and AMAC.

	Manual/ImagePro	Manual/ImagePro		AMAC	AMAC
No.	Collagen content (%)	Interstitial fibrosis (%)		Collagen content (%)	Interstitial fibrosis (%)
1	-	-		-	-
2	16,3	17,9		17,11	6,15
3	10,7	9,6		4,07	5,33
4	10,6	7,4		1,11	3,83
5	11,5	6,6		2,17	4,62
6	8	0		0,13	3,55
7	-	0		-	3,19
8	-	0		-	4,35
9	-	0		-	3,48
10	-	0		-	4,12
Average	11,42	4,61		4,91	4,29

Further, the assessment of collagen content in the scar is often separately conducted, requiring different staining and different analysis. Here, we propose the assessment of total collagen content in the scar, which should provide sufficient information for the researchers whether to continue the investigations or not. In our model, the program is set to recognize the Gomori’ one step trichrome staining and evaluating the color hue gradient of blue, from blue to gray. This is difficult to be assessed with other similar commercial software, since they cannot evaluate the threshold interval for defining each individual gradient color. The only limitation of this approach is that all required sections should be simultaneously stained, to reduce staining artifacts.

In conclusion, the development of the AMAC software showed robust simultaneous estimation of different morpho-histological parameters for serial tissue sections within seconds. AMAC enabled an increased objectivity level of image evaluation and further data processing with an increased accuracy over the conventional applications requiring manual input. Thus, the interdisciplinary structure of clinical laboratories may increase the efficiency, effectiveness, and the competitiveness of the research group, by resolving the specific problems and responding to specific needs, which cannot be solved by existent commercial software.

- **Simultaneous estimation of different morpho-histological parameters for serial tissue sections within seconds**.
- **High level of objectivity of image evaluation and data processing**.
- **The new interdisciplinary e-concept enables comprehensive correlation of relevant "in vivo" and "in vitro" parameters**.
